# Introduction to the urban ecologies open collection: A call for contributions on methods, ethics, and design in geographical research with urban animals

**DOI:** 10.1002/geo2.101

**Published:** 2021-09-28

**Authors:** Catherine Oliver, Shruti Ragavan, Jonathon Turnbull, Anmol Chowdhury, Diane Borden, Thomas Fry, Sneha Gutgutia, Shubhangi Srivastava

**Affiliations:** ^1^ Department of Geography University of Cambridge Cambridge UK; ^2^ National Institute of Advanced Studies Bengaluru India; ^3^ The University of Trans‐Disciplinary Health Sciences and Technology Bengaluru India; ^4^ Manipal Academy of Higher Education Manipal India

**Keywords:** animal geography, more‐than‐human methods, urban animals, urban justice, urban planning

## Abstract

This Open Collection proposes innovative research directions for both urban and beyond/more‐than‐/non‐human geographies with animals. We are seeking papers for this Open Collection across three themes: (1) methods; (2) ethics and politics; and (3) planning and design. Specifically, we are interested in papers that pose questions of and reflect upon emergent tensions in researching with urban animals in each of these themes. This Open Collection aims to explore urban space beyond the human lens and to offer new modalities and frameworks for geographical research with urban animals. We are interested in papers that explore urban geographies with animals from a range of different theoretical, methodological, and empirical locations and perspectives. In this introduction to the Open Collection, we briefly summarise existing research in this field, before outlining the three thematic areas of the Collection.

## Abstract 2

This Open Collection on “urban ecologies” proposes innovative research directions for both urban and beyond‐/more‐than‐/non‐human geographies, broadly construed. We are seeking papers for this Open Collection across three themes: (1) methods; (2) ethics and politics; and (3) planning and design.

## 1 INTRODUCTION

The world beyond the human has always been of concern to geographical study. The environments, landscapes, and worlds that humans live in, create, and navigate are the very basis of geographical thought. Against this backdrop, non‐human animals have overwhelmingly featured as objects of the landscape, rather than being included as subjective participants in world‐making, and research, practices. As such, geographers have argued that animals have for too long been “remote from the problems of human geography” (Davies in Philo & Wolch, 1998). As Jennifer Wolch wrote in 2002:

Geographers have long neglected the role of nature in shaping the urban experience. Yet the anima urbis – the breath, life, soul and spirit of the city – is embodied in its animal as well as human life forms. (p. 721)

In the decades since Wolch and Emel's *Animal Geographies* (1998), the idea of animals as geographical interlocutors and actors has expanded in numerous ways, most notably in the sub‐disciplines of critical animal geographies and more‐than‐human geographies. Multispecies research has become more common in geography, with multispecies contact (or conflict, Wadiwel, 2018) zones becoming a site in which geographers “have direct sensory engagement with animals, where animals become partners in our research practice” (Collard & Gillespie, 2015, p. 205). However, as these new geographies emerge, the methodologies, politics, and implications of research with non‐human animals must be visited and re‐visited. This Open Collection takes up these issues in urban space, following Amin and Thrift (2017, p. 86) who contend that cities:

Rely on organized forms of cruelty to nonhumans in order to maintain their human momentum: cities are hungry predators on other forms of life … cities have nearly always been built on the cries and screams and howls of dying animals.

Cities have been built on the exploitation of non‐human life (Thrift, 2021), but they are also sites and viable habitats for all kinds of animal life. This life might be companionable or cultivated, feral or wild, in the “biological realm” of the urban (Amin & Thrift, 2002). This biological realm of urban geography extends not only to the animal city, but to recent work on the botanical city (Gandy & Jasper, 2020) and the viral city as producing new urbanisms (see Madden, 2020). The veneer of the all‐too‐human city has been perturbed and disrupted by geographers attending to situated knowledges of the urban beyond the human. Recent work in urban geographies reveals a flourishing of more‐than‐human life in the city.

Urban political ecology (UPE) has similarly taken up the idea that nature shapes the urban experience (Heynen et al., 2006). Urban political ecologists contend that urban space extends far beyond the bounds of the city, producing a “global hinterland” (Steel, 2008). As Cornea and her colleagues argue, UPE has evolved within geography “to examine the power relations that produce uneven urban spaces (infrastructures and natures) and unequal access to resources in cities” (2017, p. 1). The city and its hinterland rely on production of what Jason Moore (2015) would call Cheap Natures: of food, labour‐power, energy and raw materials. The “Four Cheaps” are brought into cities from elsewhere (Braun, 2005), whilst hazardous waste being created in cities is dumped in remote rural areas or countries of the global South (Millington & Lawhon, 2019). Human exploitation of animals, such as slaughter, pollutes the sanitised urban space, and has increasingly become “out of place” in the human city (Philo, 1995) that prioritises capital (Atkinson, 2020).

Urban animals have captured not only the imagination of academics, but of wider publics, especially through documentary films. In the documentary series *Cities*: *Nature's New Wild* (BBC, 2018–19), the city is framed as a space of opportunity as well as danger to other species, and urban animals are explored in three categories. *Residents* make cities their permanent homes, such as a raft of otters dwelling in Singapore and the proliferation of megabats in Australian cities. *Commuters*, displaced from their ancestral habitats, access cities for shelter and food, like a herd of hippos in St Lucia, South Africa, who feast on manicured lawns. *Outcasts* compete directly with humans for space and resources, often relying on human benefactors, like swiftlets in Indonesia who have taken to living in “swiftlet hotels” in human houses. The city is, through this gaze, a contested and dangerous space that might be founded on the exploitation of other‐than‐human life, but is also filled with possibility.

Van Dooren and Rose (2012, p. 1) have similarly categorised urban animals into two groups: animals who choose to move into city spaces, and animals who find themselves overtaken or displaced by cities. While some species are new to the city, many others predate human settlement and are the descendants of the original inhabitants of these spaces (see Kean, 2011). Attending to life in the multispecies city demands constant renegotiation and consideration of more‐than‐human urban ecologies. In thinking about the place of animals in the city, geographers such as Donna Houston and her colleagues make the case that more‐than‐human perspectives on the multispecies city might “offer new possibilities for productively rethinking the ontological exceptionalism of humans” (Houston et al., 2018, p. 190) in urban theory and practice.

This Open Collection follows these lineages to call for contributions that don't only “bring the animals in” (Wolch & Emel, 1994) but use beyond‐human geographical engagements with animals to rethink urban space. As multispecies and more‐than‐human theories and approaches take up more space in the geographical canon, we contend that it is time to reflect upon the methods, ethics, and practices that are developing, as well as showcase the new modes of seeing and engaging with the city beyond the human. In this Open Collection, we welcome contributions from a range of perspectives that encompass more‐than‐human, beyond‐human, and multispecies geographies. Similarly, we welcome varying definitions and boundaries of “the urban,” especially those that reconsider or retheorise “the urban” drawing on research with urban animals.

*GEO* is distinctive in its capacity and commitment to hosting video, photographic, sound, and other enriched media formats in its contributions, and we are particularly looking for contributions that incorporate innovative formats, such as photo essays, soundscapes, films, interviews, and maps. We also invite creative responses to the contributions in the Open Collection that generate dialogue and cross‐contribution conversations.

We invite responses to three themes that are particularly salient in thinking beyond the human in urban geographies: methodological expositions; ethics, justice, and the right to the city; and urban planning, design, and infrastructure. The remainder of this introduction serves to contextualise these three themes and to detail the kinds of contributions that we are interested in receiving.

## 2 METHODOLOGICAL EXPOSITIONS

The city has long been a playground for innovative geographical methods which, for Loretta Lees (2003, p. 108), has been fundamental to educating new urban geographers in “the complexities and practicalities of method and methodology.” Without this, she claims, “the credibility of our research is at stake.” More‐than‐human and multispecies geographies not only demand new conceptualisations of the spatial scope of care (Smith, 1998), but also require researchers to move beyond anthropocentric understandings of space and community (Gibbs, 2020). In the city, more‐than‐human perspectives on urban animals recognise that “interspecies mingling is fundamental to city life” (Hovorka, 2008, p. 96) and the multitude of life forms in a city not only ﻿shape but are also “shaped by political, economic, and cultural forces” (Kirksey & Helmreich, 2010, p. 545). Thus, the posthuman turn in geographical research, which has often been concerned with ontological arguments, might also be suggestive of an epistemological and methodological turn.

Attending to the lives of nonhuman animals in urban space poses new questions and opportunities for geographical methods and methodologies (see Buller, 2015). For Urbanik (2012, p. 186), the future challenge of animal geographies lies in “developing the methodologies that will allow us to move closer to the animals themselves as individual, subjective beings.” Hodgetts and Lorimer (2015) propose a shift from animal geography to *animals’ geographies*, by developing and deploying methodologies that attend to the lived geographies of animals themselves. For Buller (2015), this presents a “triple challenge,” which entails resisting abstractions, decentring the human, and moving away from distinctions between the social and natural sciences. Finding ways for animals to not only speak but also see and be seen (Berger, 2009; Derrida, 2008) in the city allows us to reconfigure how we research with and for animals in urban spaces.

For contributions to this theme, we are looking for efforts by geographers to think not only about how we research animals, but the ways in which we undertake this: through watching, sensing, tracking, moving, smelling, and listening, for example. We envision these contributions to illustrate, through rich descriptions and critical reflexivity in flexible formats, innovative methodological practice, including and beyond the examples listed here.

## 3 ETHICS, JUSTICE, AND THE RIGHT TO THE CITY

The ethics of research with non‐human animals is part of a rich history of thinking with sentient (animal) subjects (see Hall, 2011, on the distinctions between non‐human natures and non‐human animals). Similarly, there is an established history of thinking about and with animals in politics, (political) philosophy, and law. Indeed, Cochrane et al. (2018) contend that the unifying and distinctive feature of “the political turn” in animal ethics and studies is the focus on *justice* and its importance to changing political institutions, structures and, we would add, space itself. Geographically speaking, Hobson (2007, p. 251) argues that “research which conceptualizes animals as part of, not incidental to, specific political configurations—that is, as subjects, not objects—enables a broader conceptualization of how the ‘political’ is constituted.”

The city is a space where politics unfolds in exceptional and everyday modalities, in both institutional and resistant forms. The *right to the city*, as developed by Marxist geographers and adopted by dispossessed urban groups, has since Lefebvre (1968/1996]) been conceptualised as more than an individual right, recently expanded by Shingne (2020) to include animals. As David Harvey writes, “to claim the right to the city is … to claim some kind of shaping power over the processes of urbanization, over the ways in which our cities are made and re‐made … in a fundamental and radical way” (Harvey, 2008, p. 272). Where “the urban is a highly complex field of tensions” (Lefebvre, [1970/2003], p. 40), the right to the city is one of social justice in a “politics of the commons” (Amin & Thrift, 2002).

The right to the city is not a solely human affair. Urban space is made and re‐made by a range of nonhuman actors. Recent scholarship has begun to think through displacement, justice, and the right to the *multispecies city*, accounting for animal lives in urban planning and development, for example in gentrification (Hubbard & Brooks, 2021). Considering the multispecies right to the city (Shingne, 2020) engenders novel ethico‐political challenges, demanding new conceptualisations and justifications of what Haraway (2008) might call “response‐ability” (see also Greenhough & Roe, 2010). With this in mind, “cities *can* be key testing grounds” (Amin & Thrift, 2002, p. 156) not only for research, but for expanding and reconceptualising ethico‐political questions of the urban from multispecies perspectives. Attending to multispecies justice (Celermajer et al., 2020) invites a critical rethinking of “temporal and spatial scales of eco‐social responsibility, without collapsing all of humanity into an amorphous ‘us’ or by ignoring the lively multispecies assemblies” that constitute the urban (Houston et al., 2018, p. 193).

For this section of the Open Collection, we are seeking contributions that use geographical knowledge and empirical fieldwork to trouble the ethico‐political issues in research with and alongside urban multispecies entanglements. These may revolve around questions of justice, rights, community, belonging, and care in more‐than‐human urban research with animals.

## 4 URBAN PLANNING, DESIGN, AND INFRASTRUCTURE

Traditionally, urban planning and architecture have sanitised cities, attempting to separate them from nature as exclusively human domains (Braun, 2005). Cities have been key biopolitical sites where specific inclusions and exclusions (of spaces, bodies, and practices) are enacted. In the case of nonhuman animals, exclusionary logics are visible in the form of larger urban imaginaries – such as official city planning documents (masterplans and zoning documents), regulatory mechanisms and development projects – and through everyday biopolitical acts such as, for example, stray cattle in Delhi being routinely captured, impounded and translocated to *gaushalas* (cow shelters, see Ragavan & Srivastava, 2020). In another everyday biopolitical example, for flying foxes in Australia, urban planning has intentionally fragmented their habitat, pushing them out of cities (Van Dooren & Rose, 2012). As these examples show, biopolitical control of urban space can supersede, and eliminate, co‐evolved multispecies landscapes (Houston et al., 2018), with disastrous implications for animals.

Emerging literature in urban ecologies grapples with the contradictory nature of ecological urbanism, with urban animals becoming enlisted in environmental remediation tactics and greening narratives (Houston et al., 2018). In a biopolitical reversal, nonhuman animals who were previously considered infectious, toxic, or as pests, are being re‐figured as agents of environmental sustainability. For instance, black soldier flies have been cast as metabolic labourers for circular waste economies (Zhang, 2020). Through design initiatives and infrastructural programs, non‐humans “are being encouraged to (re)colonise specific neighbourhoods” often via “deliberative human provisioning of nesting and feeding sites, such as insect hotels, beehives, hedgehog boxes and bird‐feeders” (Hubbard & Brooks, 2021, p. 7).

Animal‐centred urban infrastructures include animal bridges and highways, wildlife corridors, and green roofs, which seek to make cities more liveable for certain nonhuman inhabitants. These speculative design strategies can foster novel forms of co‐design (Ávila & Ernstson, 2019) and new cohabitations with urban animals. As urban green space increasingly falls under the techno‐managerialist purview of urban planning, non‐human animals have become subjects of, and enrolled within, wider political‐ecological projects that seek to construct smart, green, or resilient cities, and their associated patterns of gentrification and spatial exclusion (Anguelovski et al., 2019). Even with the emergence of beyond‐human designs, there remain questions of who can flourish under these new configurations of urban co‐habitation, and who cannot. As Barua (2021) contends, infrastructure not only shapes animals’ mobilities and atmospheres, but also how animals themselves can become urban infrastructure.

Geographers have a vital role in understanding how nonhuman animals *use* and *make* urban spaces. We are looking for rich empirical contributions that draw together empirical work with critical visions of the urban, such as design, architecture, infrastructures, governance, more‐than‐human commoning, hybridity, and futurity.

## 5 CONCLUSION

Geographers are increasingly approaching the urban through a more‐than‐human lens, constituting a “dizzying series of theoretical, philosophical and methodological transformations” (Braun, 2005, p. 635). However, these urban stories are often subsumed to questions around the human city. How we live with urban animal residents remains marginal to larger city stories. In this Open Collection, we are interested in contributions that go further than telling stories of animals in urban spaces; we are interested in work that prises open, reframes, and thinks the city anew with and for urban animals. Across the three themes of this collection – Methods, Ethics, and Planning – we are seeking geographical contributions from a range of perspectives with any species or individual animals in any city. We will be particularly interested in contributions that showcase new ways of thinking and approaches to animals in urban spaces that rethink geographic theory and practice from perspectives beyond the human. Finally, we are committed to including and prioritising innovative formats in this Open Collection, such as video, photography, sound, artwork, and other creative outputs, complementing or supplementing traditional academic writing. By engaging with urban animals in this broader range of mediums, we aim to develop and showcase new ways of representing animals in research outputs.

6

**VIDEO 1:** Street dogs in Delhi showing their everyday relations of care and affect through which they make their space in the city Methods GEO SS.mp4

**VIDEO 2:** Chickens pecking the ground searching for worms Ethics CO.mp4

**VIDEO 3:** Macaques rethinking urban infrastructures Video GEO AC.mp4

## Data Availability

Data sharing not applicable to this article as no datasets were generated or analysed.
