# Supplementary material for: Introduction to the urban ecologies open collection: A call for contributions on methods, ethics, and design in geographical research with urban animals
Source: Geo. 2021 Sep 28;8(2):e00101. doi: 10.1002/geo2.101 (PMC8518968; doi:10.1002/geo2.101)
Supplement: Supplementary file 1 [file GEO2-8-e00101-s001.pdf]

Call for contributions: Urban Ecologies Beyond the Human

An Open Collection to be hosted in *Geo: Geography and Environment*

Abstract

This Open Collection proposes innovative research directions for urban and beyond-/more-than-/non-human geographies, broadly construed. We are seeking papers for this Open Collection across three themes: (1) methods; (2) ethics and justice; and (3) planning and design. We are interested in papers that pose questions of and reflect upon emergent tensions within this field through expositional papers. This Open Collection aims not only to explore urban space beyond the human lens, but also to offer new modalities and frameworks for researching, thinking with, and practicing geographical research in multispecies urban worlds.

Instructions for contributors

This is an open call, meaning submissions are welcomed from anyone working on themes related to the topic. Contributors are welcome to contact either Dr Catherine Oliver (co419@cam.ac.uk) or the journals office (journals@rgs.org) to discuss their submission, however they are also welcome to just submit directly to the journal.

All submissions should adhere to the journal's guidelines (available at: https://rgs-ibg.onlinelibrary.wiley.com/page/journal/20544049/homepage/forauthors.html). Geo has no specific word limits for outputs, however we would suggest between 2,000 and 10,000 words for submissions. Contributors should select the option 'open collection' when submitting and give the title '*Urban Ecologies Beyond the Human'*. There are three themes covered by the Open collection: (1) methodological expositions; (2) ethics, justice, and the right to the city; and (3) urban planning, design, and infrastructure. Please specify which theme(s) you see your contribution sitting within at the point of submission. All contributions will be peer reviewed.

Geo is also capable of hosting multimedia files (video and sound) directly within the article – rather than as supplementary materials – and we strongly urge authors to make use of these capabilities within their articles. We are enthusiastic about making full use of these various media, particularly around topics that exceed language-based analysis, such as within beyond-human geographies.

The call will remain open until **1 April 2023** and we will accept first submissions up until that date. Revised submissions can still be submitted after this deadline. If your paper is not selected for inclusion in the Open Collection, it will still be considered as a regular paper for the journal.

Please note that Geo is an Open Access journal and therefore charges APC fees. For more information on these fees and the many discounts and exemptions which apply, please visit: https://rgs-ibg.onlinelibrary.wiley.com/page/journal/20544049/homepage/custom_copy.html

Workshops

The convenors of this open call are keen to support authors and the wider community of scholars working in this area. Authors who are interested in submitting a paper to the open collection will be invited to online workshops hosted by the Urban Ecologies group at the University of Cambridge. There will be three workshops, one related to each theme, hosted between 2021 and 2023. These workshops are not compulsory for inclusion in the open collection, however, if you are interested in taking part in these, please contact Dr Catherine Oliver (co419@cam.ac.uk).
